# Psychedelic iatrogenic structural dissociation: an exploratory hypothesis on dissociative risks in psychedelic use

**DOI:** 10.3389/fpsyg.2025.1528253

**Published:** 2025-03-04

**Authors:** Steven Elfrink, Leigh Bergin

**Affiliations:** ^1^OmTerra, Oregon, OR, United States; ^2^Department of Psychology, University of Exeter, Exeter, United Kingdom

**Keywords:** psychedelic therapy, trauma, dissociation, structural dissociation, dissociative symptoms, adverse effects, trauma-informed care, psychedelic iatrogenic structural dissociation

## Abstract

This paper introduces the hypothesis of Psychedelic Iatrogenic Structural Dissociation (PISD), proposing that psychedelics may reactivate dissociated traumatic material, increasing the risk of psychological destabilization in trauma-exposed individuals. Grounded in structural dissociation theory, this framework suggests that psychedelics can disrupt the balance between daily functioning (the Apparently Normal Personality, ANP) and trauma-related responses (the Emotional Personality, EP), leading to the resurfacing of unintegrated memories. A review of recent studies highlights persistent adverse effects associated with psychedelic use, including emotional dysregulation, identity fragmentation, derealization, and perceptual disturbances, particularly among individuals with early trauma histories. To mitigate these risks and facilitate trauma processing, integration practices, body-focused therapies, and structured social support systems are proposed as key interventions. Additionally, emerging neurophysiological models suggest that psychedelics may reconfigure dissociative processes through the modulation of entrenched patterns, potentially facilitating trauma integration or increasing vulnerability to destabilization, depending on individual and contextual factors. These findings underscore the necessity of trauma-informed screening, preparation, and integration protocols to enhance the safety and efficacy of psychedelic therapies, particularly for vulnerable populations.

## Introduction

The phenomenon of dissociation has long captivated clinicians, researchers, and scholars in psychology and psychiatry (Van der Hart et al., 2005; Loewenstein, 2018). Defined as a disruption, interruption, and/or discontinuity of the normal, subjective integration of behavior, memory, identity, consciousness, emotion, perception, body representation, and motor control, dissociation manifests in various forms and degrees of severity (American Psychiatric Association, 2013; Loewenstein, 2018). The Diagnostic and Statistical Manual of Mental Disorders, fifth edition (DSM-5), categorizes dissociation under dissociative disorders (DD) and describes more severe and clinically significant forms. These include Dissociative Identity Disorder (DID), Dissociative Amnesia (DA), Depersonalization/Derealization Disorder (DPDRD), Other Specified Dissociative Disorders (OSDD), and Unspecified Dissociative Disorder (UDD) (American Psychiatric Association, 2013; Loewenstein, 2018).

While the DSM-5 provides a diagnostic framework for dissociative phenomena, it is not without limitations. By focusing primarily on pathological forms of dissociation, it overlooks the broader spectrum, which includes “normal” dissociative experiences that are often adaptive and universal (Loewenstein, 2018). Examples include daydreaming, absorption in activities, or “zoning out” while driving—non-pathological interruptions of consciousness that reflect dissociation’s functional versatility (Waller et al., 1996). Similarly, processes like code-switching—a linguistic and cultural adaptation where individuals shift between modes of communication based on context—highlight dissociation’s role in facilitating adaptive responses to environmental demands (Javier and Marcos, 1989). A related phenomenon, known as swift switching, refers to the cognitive ability to transition rapidly between mental representations and has been proposed as a mechanism underlying pathological dissociation, particularly in trauma-exposed individuals (Chiu et al., 2016). This “trauma-related switching hypothesis” suggests that childhood traumatisation may enhance switching abilities, contributing to dissociative coping mechanisms in clinical populations (Chiu et al., 2016). Together, these concepts illustrate the mind’s capacity to flexibly adapt to changing contexts, a capacity that, when disrupted by trauma, may become dysregulated and lead to pathological dissociation.

Additionally, the Internal Family Systems (IFS) model conceptualizes dissociation as a natural division of the psyche into “parts,” which can function adaptively or maladaptively depending on context (Schwartz, 1995). According to this model, the mind comprises discrete subpersonalities—such as exiles, managers, and firefighters—each playing distinct roles within an internal system. Trauma disrupts this harmony, forcing parts into extreme roles and fragmenting the psyche. For instance, exiles, associated with repressed traumatic memories, become isolated to protect the system from overwhelming emotions, while managers and firefighters adopt hypervigilant or impulsive behaviors to shield the Self from harm. This systemic perspective complements the DSM’s focus on pathological dissociation by illustrating how dissociation operates on a spectrum. It also underscores dissociation’s capacity to reflect mechanisms of resilience, creativity, and identity negotiation, particularly when trauma-informed therapeutic approaches help restore balance among internal parts (Schwartz, 1995; Pais, 2009).

Debates and controversies have surrounded the nature, etiology, and treatment of dissociative phenomena since their systematic description in the early 19th century (Loewenstein, 2018). Central to these debates is the relationship between dissociation and trauma. While substantial evidence supports a trauma-related etiology for dissociative disorders, some argue that dissociation is linked to imaginative processes, where individuals generate trauma-related fantasies without actual trauma exposure (Giesbrecht et al., 2008; Loewenstein, 2018). In contrast, other researchers emphasize the risk of iatrogenic dissociation—conditions potentially arising from therapeutic influence and characterized by confabulated trauma memories (Brand et al., 2014).

In response to these debates, this paper introduces the hypothesis of Psychedelic Iatrogenic Structural Dissociation (PISD) as a framework for understanding dissociative risks and opportunities in psychedelic therapy. Grounded in the trauma model of structural dissociation, the PISD hypothesis explores how psychedelics might interact with and amplify latent dissociative material as a protective mechanism, particularly in trauma-exposed individuals (van der Hart et al., 2004; Thal et al., 2021). This amplification, we suggest, may have the potential to exacerbate fragmentation, leading to structural dissociation, or, in trauma-informed therapeutic contexts, present opportunities for trauma integration (Grof, 1985; Van der Hart et al., 2004; Van der Hart et al., 2006; Nijenhuis et al., 2010; Grof, as cited in Stolaroff, 2022). Emerging neurophysiological models provide further insights into how psychedelics might modulate entrenched dissociative patterns, either promoting integration or risking destabilization, depending on the context and support provided (Carhart-Harris et al., 2019; Carhart-Harris et al., 2023).

While some clinicians express concerns about potential psychiatric and neurocognitive risks, a majority report openness to incorporating psychedelic therapy into their practices if approved, as evidenced by recent surveys highlighting positive shifts in attitudes among psychiatrists and psychologists (Barnett et al., 2018; Davis et al., 2022; Barnett et al., 2024a). Given the clinical community’s increasing embrace of psychedelics for their therapeutic potential, a rigorous evaluation of dissociative risks and trauma-informed strategies to support the safe and effective administration of psychedelics becomes imperative (Smith et al., 2022; Ko et al., 2023). Due to the unpredictable nature of these effects and their potential resistance to conventional protective measures, this paper advocates for advanced screening, preparatory assessments, and personalized integration practices to mitigate risks. The following section examines the trauma model of dissociation to contextualize how trauma influences personality integration and informs the analysis of psychedelics’ effects on dissociative processes.

### The trauma model of dissociation

The trauma model of dissociation offers a comprehensive framework for understanding dissociative phenomena in relation to psychological trauma (Loewenstein, 2018). According to this model, dissociation functions as a psychobiological state or trait that serves as a protective mechanism, helping individuals to distance themselves from overwhelming emotions and sensory experiences linked to trauma (Van der Kolk, 2000; Van der Kolk, 2014). A substantial and growing body of evidence underscores the strong relationship between dissociation and psychological trauma, particularly when trauma is cumulative or occurs early in life (Briere and Scott, 2006).

As mentioned, dissociation is often viewed as a spectrum, ranging from normal experiences—such as deep absorption in activities like reading, writing, or driving—to more pathological states, including severe dissociative disorders (Csikszentmihalyi, 1975; Loewenstein, 2018). An alternative conceptualization, known as the Taxon Model, posits two separate continua: one for normal dissociative experiences and another for pathological dissociation (Putnam, 1991). In this model, a distinct subgroup of highly traumatized individuals—comprising approximately 3.5% of the general population—demonstrates a cluster of symptoms indicative of severe dissociative psychopathology, such as depersonalization, recurrent amnesia, and identity alterations (Dell and O'Neil, 2010; Loewenstein, 2018).

The trauma model suggests that dissociation functions as a defense mechanism, mitigating the impact of trauma by sequestering traumatic memories within altered states of consciousness (Van der Hart et al., 2006). Through this compartmentalization, individuals can distance themselves from the full emotional impact of trauma, which aids in coping with otherwise overwhelming experiences. Treatment approaches grounded in the trauma model emphasize safety and stabilization, focusing on immediate concerns such as self-injurious behaviors, suicidal ideation, and the management of dissociative states before directly addressing traumatic memories (Loewenstein, 2018).

Several alternative models challenge the trauma model’s premise that dissociation is primarily a protective response to trauma (Loewenstein, 2018). One such model, the Iatrogenic Model (IM), posited by Paris (2012), suggests that DID is a “medical fad” artificially induced during treatment. However, this perspective has been widely criticized for its reliance on outdated sources and dismissal of robust neurobiological and clinical evidence (Brand et al., 2013). For instance, neuroimaging studies reveal brain abnormalities in DID patients consistent with trauma-related disorders, countering claims that DID lacks a biological basis (Vermetten et al., 2006). Additionally, large-scale meta-analyses demonstrate a dose–response relationship between early trauma, DD, post-traumatic stress disorder (PTSD), and other outcomes, while phase-oriented approaches to DID therapy are associated with significant reductions in dissociation, PTSD, depression, and self-harm, further supporting trauma’s centrality in dissociation’s etiology (Felitti and Anda, 2010). Critics emphasize that dismissing DID as a “fad” trivializes the experiences of highly traumatized individuals and contributes to stigma in both clinical and public domains (Brand et al., 2013; Loewenstein, 2018).

Other models, such as the Sociocognitive Model (SCM) and Fantasy Model (FM), emphasize sociocultural or cognitive mechanisms over trauma (Lilienfeld et al., 1999; Giesbrecht et al., 2008; McHugh, 2008). The SCM posits that exposure to cultural narratives about dissociation can lead to the adoption of dissociative behaviors among vulnerable individuals, while the FM suggests that imaginative processes generate trauma-related fantasies. Proponents of the trauma model, however, highlight its superior predictive validity in explaining symptom trajectories, therapy responses, and trauma–dissociation relationships (Dalenberg et al., 2012). Importantly, these models may not be mutually exclusive and may offer complementary insights into how cognitive and sociocultural factors interact with trauma to shape dissociative manifestations. That said, substantial empirical evidence continues to support the trauma model’s relevance, particularly in trauma-focused therapies that prioritize stabilization and symptom management over direct memory recovery (Brand et al., 2014). The trauma model’s foundations remain deeply rooted in early psychiatric research and historical observations linking trauma to dissociative symptoms (Hustvedt, 2012; Loewenstein, 2018).

#### Historical research of dissociative disorders

DD are among the oldest documented psychiatric conditions, first appearing in medical literature in the late 18th century (Bogousslavsky, 2015; Loewenstein, 2018). Throughout the 19th century, physicians such as Charcot and Janet began systematically investigating dissociative phenomena, identifying psychological and post-traumatic factors as key contributors (Janet, 1894; Van der Hart and Friedman, 1989). Their work laid the foundation for the trauma model, emphasizing trauma as central to the development of dissociative symptoms. Janet, in particular, advanced a framework closely resembling contemporary understandings of trauma and dissociation, foregrounding the impact of early life trauma, abuse, neglect, and social marginalization in shaping dissociative psychopathology. He argued that trauma’s dissociative effects are proportionate to its severity, including factors such as intensity, duration, and repetition (Van der Hart and Friedman, 1989; Loewenstein, 2018).

Research into medical records from La Salpêtrière, where Charcot and Janet conducted much of their work, supports the trauma model (Van der Hart and Friedman, 1989; Loewenstein, 2018). Many patients treated there had endured significant trauma, including sexual and physical abuse, accidents, traumatic losses, and social adversity (Hustvedt, 2012; Walusinski, 2014). Furthermore, exposure to wartime trauma likely contributed substantially to the psychopathology observed in these patients, as dissociative symptoms have frequently been reported in individuals affected by the traumas of war. Studies have documented high rates of dissociative amnesia, fugue states, depersonalization/derealization, and other dissociative symptoms among soldiers post-conflict (Carlson and Rosser-Hogan, 1991). Similarly, survivors of atrocities, such as the Holocaust, and victims of torture exhibit elevated levels of dissociative symptoms (Kuch and Cox, 1992; Van Ommeren et al., 2001; Loewenstein, 2018). As the study of dissociation has evolved, modern research methodologies have facilitated a more systematic investigation of DD, identifying consistent patterns across diverse populations.

#### The scientific study of dissociation

Since the 1980s, researchers have developed reliable and valid tools to assess both state and trait dissociation, as well as DD (Loewenstein, 2018). Studies across diverse clinical and general populations consistently identify individuals with DD, with findings linking higher dissociation scores and diagnoses to acute or chronic trauma (Spiegel et al., 2011). Retrospective and prospective studies across cultures further demonstrate a clear correlation between trauma severity and dissociative symptoms, with more severe trauma generally associated with higher dissociation scores on standardized measures (Spiegel et al., 2011; Loewenstein, 2018).

Longitudinal research indicates that early and cumulative trauma, as well as childhood attachment issues, predict elevated dissociation levels and the later development of DD (Teicher et al., 2016). For example, one study found that in high-risk children, early dissociative symptoms were closely associated with trauma severity and disorganized attachment patterns, predicting dissociative symptoms up to two decades later (Granqvist et al., 2017). Additionally, peritraumatic dissociation has been identified as a predictor of PTSD, DD, and somatoform disorders, underscoring dissociation’s central role in trauma-related psychopathologies (Marmar et al., 1996; Darves-Bornoz, 1997; Ogawa et al., 1997; Shalev et al., 1998).

### The trauma theory of structural dissociation

After traumatic events, individuals may oscillate between reliving the experience and distancing themselves from its impact, a fluctuation that involves distinct mental states (Van der Hart et al., 2004; Nijenhuis et al., 2010). The trauma theory of structural dissociation suggests that severe or early-life trauma activates deeply ingrained emotional and functional systems known as “action systems,” each with its own sense of self, which may remain fragmented or unintegrated (Fanselow and Lester, 2013; Panksepp, 2004). According to this theory, trauma induces a division within the personality, separating systems involved in daily functioning from those oriented toward survival and defense. This division, termed *primary structural dissociation*, creates a split between the “apparently normal” personality (ANP) and the “emotional” personality (EP) (Van der Hart et al., 2004).

Building on Janet’s (1889) early work, Nijenhuis et al. (2010) conceptualize mental health as dependent on the capacity for differentiation and synthesis. Synthesis refers to the integration of internal and external experiences into coherent mental structures, enabling adaptive responses to challenges (Braude, 1995). A critical component of this synthesis is *personification*, the process by which individuals relate experiences to their self-identity (Janet, 1928). Through personification, individuals contextualize trauma within their broader life narrative, facilitating a cohesive sense of self. However, overwhelming events can disrupt this integration, leaving trauma as an unprocessed semantic memory rather than a self-reflective episodic memory, thereby impeding the continuity of one’s identity over time (Tulving, 1993; Tulving, 2002).

Peritraumatic dissociation occurs when individuals cannot fully integrate a severely threatening event (Marmar et al., 1996; Nijenhuis et al., 2010). This failure to synthesize may present as feelings of unreality, out-of-body experiences, bodily disconnection, analgesia, or impaired motor responses (Nijenhuis, 2001). Central to peritraumatic dissociation is the failure of personification, whereby individuals recognize the threat but do not internalize it as part of their personal experience. This phenomenon is especially significant in young children, who are particularly vulnerable to dissociative experiences during or after trauma. Research consistently links peritraumatic dissociation to long-term trauma-related disorders (Boon and Draijer, 1993; Kluft, 1993; Putnam, 1997; Van der Hart et al., 2005).

Empirical studies indicate that peritraumatic dissociation may predict later development of PTSD and DD, though the predictive value of dissociation in acute stress disorder remains debated (Shalev et al., 1996; Nijenhuis and Den Boer, 2009). Some research suggests that trauma re-experiencing is a stronger predictor of PTSD than dissociation, though Nijenhuis et al. (2010) maintain that trauma re-experiencing is itself a symptom of dissociation, tied to the incomplete synthesis and personification of traumatic memories (Harvey and Bryant, 1999). Drawing on Myers (1940) foundational ideas, Nijenhuis et al. (2010) propose that *primary structural dissociation* creates a division within the personality, with trauma-related memories and reactions (EP) remaining distinct from systems managing daily life (ANP). In more complex cases, this dissociation can progress to *secondary* or *tertiary* forms, further fragmenting the personality and complicating recovery (Nijenhuis et al., 2010).

#### The emotional personality

Nijenhuis et al. (2010) describe the EP as a mental system that stores traumatic memories. These memories are *autonoetic*, meaning they are experienced with an immediate self-referential awareness, as though the events are actively occurring (Panksepp, 2004). EP memories encapsulate the core aspects of trauma, representing isolated incidents and the essential elements of overwhelming events. Unlike processed trauma narratives, EP memories are fixed and nonverbal, consisting of sensory and perceptual phenomena such as visual images and sensations, as well as physical responses like defensive motor reactions (Janet, 1928; Van der Kolk and Van der Hart, 1991). These experiences are often tied to an alternative bodily image and a distinct sense of self, manifesting in various forms—from re-experiencing unprocessed trauma in acute PTSD to fragmented dissociative parts in DID (Van der Kolk, 2000; Nijenhuis et al., 2010).

EP memories differ from processed memories in that they lack adaptability and remain experientially bound to the past, without a sense of social function or temporal continuity (Janet, 1928; Van der Kolk and Van der Hart, 1991). Typically, EPs are fixated on past traumatic events, responding to perceived threats with basic defensive reactions and limited awareness of present circumstances (Janet, 1928; Van der Hart and Steele, 1997; Nijenhuis and Den Boer, 2009). While EP memories reflect core aspects of the original trauma, they are not exact replications; instead, they often contain distortions, such as fantasies or omissions, which further complicate the memory. During reactivation, EP memories can inhibit access to other forms of memory (e.g., episodic, semantic, procedural) that are typically accessible to the ANP, thereby intensifying the sense of immediacy (Van der Hart and Nijenhuis, 2001; Van der Hart et al., 2001; Nijenhuis et al., 2010).

Defensive behaviors associated with the EP, such as immobility, hiding, or protective posturing, are typically triggered by conditioned trauma cues (Nijenhuis et al., 2010). However, these responses may subside in safe environments. For example, an EP representing a childlike self-state in cases of childhood abuse may occasionally express playfulness when no threat is perceived (Putnam, 1997; Van der Hart and Nijenhuis, 2001). Research supports the hypothesis that traumatic memories within the EP are predominantly sensorimotor, often recalled as sensory and affective imprints with minimal verbal content (Van der Kolk and Fisler, 1995; Nijenhuis et al., 2010). For instance, sexually abused children may encode and retrieve trauma primarily through sensory perceptions and behavioral responses (Burgess et al., 1995; Van der Kolk and Fisler, 1995; Nijenhuis, 2001). In cases of DID, EPs may selectively attend to aspects of the present that resemble past trauma, but remain unable to fully integrate current reality, thereby limiting their adaptive potential (Nijenhuis et al., 2010).

#### The apparently normal personality

While the EP encapsulates unprocessed trauma, the ANP enables individuals to engage in daily life, albeit often with psychological disruptions (Nijenhuis et al., 2010). Operating within the ANP, individuals may either partially integrate or completely disconnect from their trauma, creating a functional illusion of normalcy. However, this division between trauma and day-to-day consciousness often incurs a psychological cost. The ANP is characterized by dissociative symptoms, including amnesia, sensory anesthesia, and depersonalization, which detach the emotional impact of traumatic events from conscious awareness. Lacking the process of personification, the ANP leaves traumatic experiences emotionally distant and disconnected (Nijenhuis et al., 2010).

The level of functionality within the ANP varies considerably (Nijenhuis et al., 2010). For instance, individuals with PTSD or DID may maintain daily routines and succeed in personal or professional life for extended periods (Horevitz and Loewenstein, 1994). Others, however, may experience severe amnesia or a marked decline in functioning, limiting their ability to cope with everyday demands (Nijenhuis, 2001). Janet (1911) observed that trauma could fragment the personality, impeding its development and creating gaps that hinder the integration of new experiences. This fragmentation traps individuals in a superficial state of consciousness, disconnected from deeper personality layers, including the EP (Janet, 1919; Apelfeld, 1994).

Although the ANP may appear stable, its functionality is often fragile and prone to disruption by intrusions from the EP, particularly through traumatic memories (Nijenhuis et al., 2010). These memories generally remain in a dispositional state—a latent tendency that does not manifest immediately but can surface suddenly, consuming mental and emotional resources upon activation (Charcot, 1992; Janet, 1894; Van der Hart et al., 1993). Once triggered, the ANP may experience fragmented memories, sensations, or even perceive the EP’s voice. These intrusions, while unsettling, are considered positive symptoms due to their intrusive quality (EP activation). However, they can also manifest as negative symptoms (ANP deactivation), such as inhibited movement or amnesia of the event.

The interaction between EP and ANP is dynamic, regulated by complex psychobiological systems that govern behavior and emotional responses (Nijenhuis and Den Boer, 2009). These “action systems,” associated with distinct areas of the central nervous system, are central to modulating shifts between trauma-related responses and normal functioning (Panksepp, 2004; Nijenhuis et al., 2010).

#### Action systems as regulators of EP and ANP interaction

Action systems function as self-organizing and self-stabilizing mechanisms that help maintain equilibrium across physiological and psychological states (Fanselow and Lester, 2013; Panksepp, 2004; Nijenhuis et al., 2010). Emerging early in life, they develop stable traits while allowing for considerable variation shaped by individual history and biological predispositions. Action systems integrate sensory, cognitive, and behavioral responses, facilitating adaptive or maladaptive dissociative states in response to trauma (Panksepp, 2004; Nijenhuis et al., 2010).

Panksepp (2004) noted that emotional operating systems have evolved to produce coordinated effects, influencing both higher-level cognitive functions and subcortical brain processes. Organized at a subcortical level, these systems generate integrated emotional responses through distinct patterns of brain activation. For example, action systems responsible for defensive behaviors, such as freezing or fighting, synchronize physiological and psychological states into cohesive defensive responses. This synchronization allows them to manage both trauma re-experiencing and normal functioning, as seen in the roles of the EP and ANP (Panksepp, 2004; Nijenhuis et al., 2010).

These systems are highly susceptible to classical conditioning, whereby environmental or emotional triggers can activate trauma-related responses encoded within the EP (Fanselow and Lester, 2013; Nijenhuis et al., 2010). Action systems governing attachment, defensive behavior, and parental care are particularly intertwined with trauma processing and response to external threats (Panksepp, 2004). This helps explain how specific triggers, such as sensory cues or emotional states, can cause shifts between ANP functioning and EP trauma reliving. Additionally, action systems exhibit adaptive, rather than purely reflexive, responses (Nijenhuis et al., 2010). For example, while the flight response is defensive, it involves coordinated, context-dependent actions—such as adjusting speed and direction in response to the perceived threat (Fanselow and Lester, 2013). These systems regulate defensive responses through an integrated process that involves perception, emotion, memory, and behavior, forming adaptive survival patterns.

Threats activate distinct phases within the defensive system: pre-encounter (heightened arousal), post-encounter (flight, freeze, fight), and post-strike (submission) (Fanselow and Lester, 2013; Nijenhuis et al., 2010). For example, the freeze response may involve analgesia and anesthesia, while the submission phase enables recuperation and a return to routine behaviors, such as feeding or nurturing offspring (Panksepp, 2004). Each phase corresponds to a specific subsystem within the broader defense system. Early emotional experiences can reconfigure these action systems, shaping brain function and structure over the lifespan (Panksepp, 2004).

Nijenhuis and Den Boer (2009) identify five key criteria that action systems must meet to account for the psychobiological dynamics between the ANP and EP in structural dissociation:

1. Self-organizing and self-stabilizing: Action systems maintain internal equilibrium within specific parameters of homeostasis, time, and context, thereby stabilizing the coherent psychobiological patterns exhibited by the ANP and EP.
2. Evolutionary function: Action systems are foundational, evolutionarily developed mechanisms, similar to those found in animals. Clinical observations indicate that ANPs engage primarily in tasks essential for survival, such as reproduction, attachment, socialization, and avoidance of trauma-related memories.
3. Susceptibility to conditioning: These systems respond robustly to conditioned threat cues, as both ANP and EP are highly reactive to such cues.
4. Stability with flexibility: Action systems exhibit stable traits but also adapt based on specific circumstances, mirroring the stable yet variable characteristics of ANP and EP.
5. Early development: These systems emerge early in life, a prerequisite for the manifestation of DD, which often arise in early childhood.

##### The EP and survival mechanisms in response to threat

The EP is fundamentally geared toward survival, particularly when responding to significant threats (Nijenhuis et al., 2010). This aspect of the personality is governed by action systems that manage both defense mechanisms and attachment behaviors essential for survival. Although EPs are influenced by genetic predispositions, their specific characteristics are shaped by environmental factors, particularly early childhood trauma. Critical determinants, such as the availability of social support and the recurrence of traumatic events, profoundly affect the EP’s development. In cases of primary structural dissociation, such as acute stress disorder or simple PTSD, the EP organizes various defensive responses within a unified subsystem (Nijenhuis et al., 2010).

Structural dissociation theory posits that the chronicity and severity of trauma amplify the division between the EP and the ANP, with the extent of this division contributing to the manifestation of complex trauma-related disorders (Van der Hart et al., 2005; Fung et al., 2023). In more complex conditions, such as DD or complex PTSD (cPTSD), secondary structural dissociation leads to further fragmentation, with different parts of the EP adopting specialized defensive roles, such as freezing, resisting, or submitting. Dissociation can also manifest sequentially—where distinct responses activate in stages—or in parallel, where one part of the EP observes the trauma while another directly experiences it (Nijenhuis et al., 2010). These dissociative processes are compounded by phobic avoidance of trauma-related memories and feelings, contributing to the hallmark affective dysregulation and relational difficulties observed in cPTSD (Van der Hart et al., 2005; Nester et al., 2022; Fung et al., 2023).

Recent research highlights dissociation as a core feature of cPTSD, with up to up to 76.9% of patients experiencing clinically significant levels (Haselgruber et al., 2021; Fung et al., 2023). In cPTSD, dissociation manifests through both psychoform (e.g., flashbacks, derealization) and somatoform symptoms (e.g., numbness, motor impairments), reflecting the EP’s survival-oriented responses in the development of the condition. Here, dissociation acts as both a protective adaptation and a barrier to integration, perpetuating unresolved trauma that continues to impair functioning. Notably, dissociation has also been shown to mediate the relationship between childhood trauma and cPTSD symptoms (Van Dijke et al., 2018; Fung et al., 2023).

Interventions targeting dissociation may significantly improve treatment outcomes for cPTSD. Approaches focusing on reducing “dissociative phobia” and strengthening integrative capacities have shown promise (Van der Hart et al., 2005; Steele et al., 2016). Techniques such as mindfulness, which counteract dissociation by promoting awareness and emotional processing, have been proposed as adjunctive strategies for managing cPTSD symptoms (Forner, 2019).

##### The ANP and daily life management: survival mechanisms and attachment dynamics

While the EP is oriented toward survival in response to threats, the ANP manages tasks essential for daily life and species survival, such as eating, sleeping, caregiving, and exploration (Nijenhuis et al., 2010). These tasks are regulated by specific emotional operating systems, particularly those related to attachment, social cooperation, and reproduction—all central to human survival and development (Panksepp, 2004).

A critical component within this framework is the attachment system, particularly in caregiving roles. Individuals with structural dissociation may struggle to fully personify the role of a parent, resulting in emotional detachment or numbing (Nijenhuis et al., 2010). For example, an ANP experiencing bodily anesthesia might interact with a child in a detached, mechanical manner, lacking emotional connection, which can adversely affect the child’s emotional development. This emotional disconnection can disrupt caregiving, straining the parent–child bond. In cases where the caregiver is also a source of threat, the mind may split these conflicting drives across distinct parts of the personality: the ANP maintains attachment to the caregiver, while the EP manages the defensive response to the perceived danger (Liotti, 1992).

Research indicates that early trauma, particularly from primary caregivers, often results in disorganized or avoidant attachment patterns (Liotti, 1992; Main and Morgan, 1996). Longitudinal studies show that disorganized attachment in childhood, combined with the severity and chronicity of abuse, predicts dissociation in later development (Ogawa et al., 1997; Schuengel et al., 1999). Thus, the interplay between attachment and defense mechanisms contributes to structural dissociation, as the ANP and EP form to reconcile conflicting needs for safety and connection (Nijenhuis et al., 2010).

In more complex cases, secondary structural dissociation occurs, with further fragmentation of the EP into parts that serve distinct defensive roles. In cases of tertiary structural dissociation, found primarily in DID, both the ANP and EP may divide further, resulting in multiple ANP and EP parts (Van der Hart et al., 1998; Nijenhuis and Van der Hart, 1999). Tertiary dissociation typically arises not at initial traumatization but later, as daily life becomes inextricably linked to past traumatic experiences, often due to the overgeneralization of stimuli that trigger traumatic memories (Nijenhuis and Den Boer, 2009). This level of dissociation may result from early, chronic traumatization that impedes cohesive personality development, leading to a complex configuration of ANP and EP with blurred boundaries (Nijenhuis and Den Boer, 2009). As in secondary structural dissociation, the degree of fragmentation depends on various factors, including the severity, chronicity, and intensity of trauma; the developmental age at onset; the nature of the relationship with the perpetrator(s); and the availability—or lack—of social support and recognition of the traumatic experiences (Nijenhuis and Den Boer, 2009).

### The resurgence of psychedelic research

Psychedelics represent a class of psychoactive substances known for their ability to induce non-ordinary states of consciousness, with profound effects on perception, cognition, emotion, and self-concept, commonly referred to as “ego” (Freud, 1895; Epstein, 1988; Nichols, 2016; Swanson, 2018). Among the most well-known psychedelics are “classic” serotonergic compounds, which primarily exert their effects through activation of serotonin 2A receptors (Vollenweider et al., 1998; Preller et al., 2018). This category includes psilocybin (4-phosphoryloxy-N,N-dimethyltryptamine), a principal component of *psilocybe* mushrooms and precursor to its psychoactive metabolite, psilocin (4-hydroxy-N,N-dimethyltryptamine) (Hofmann et al., 1958; Nichols, 2020). Other prominent classic psychedelics include LSD (lysergic acid diethylamide), DMT (N,N-dimethyltryptamine)—the primary psychoactive constituent of the Amazonian brew ayahuasca—and 5-MeO-DMT (5-methoxy-N,N-dimethyltryptamine), found in various plant species and in the glands of the Colorado River toad (*Incilius alvarius*) (Dobkin, 1968; Bussmann and Sharon, 2006; Socha et al., 2022). Additionally, mescaline (3,4,5-trimethoxyphenethylamine) is derived from culturally significant cacti, such as peyote (*Lophophora williamsii*) and San Pedro (*Trichocereus pachanoi*) (Stewart, 1987; Halpern, 2004). With the exception of LSD, first synthesized in 1938, classic psychedelics have been used culturally for millennia, with deep historical roots in various civilizations, particularly among indigenous peoples in South and Central America (Hofmann, 1979; Halpern, 2004; Guzmán, 2008).

Within the broader spectrum of psychedelics are dissociatives, such as ketamine, PCP (phencyclidine), nitrous oxide, and dextromethorphan. These substances act as N-methyl-D-aspartate (NMDA) receptor antagonists, disrupting normal glutamatergic neurotransmission (Anis et al., 1983). Another category includes empathogen-entactogens, exemplified by MDMA (3,4-Methylenedioxymethamphetamine), which primarily function as substrates for monoamine vesicular transporter proteins (Nichols, 1986).

Atypical psychedelics present unique pharmacological profiles. For instance, salvinorin A, a kappa-opioid receptor agonist, is the active compound in *Salvia divinorum*, a plant traditionally used by the Mazatec people of Oaxaca, Mexico (Roth et al., 2002; Hernández-Alvarado et al., 2020). Another example is ibogaine, an indole alkaloid from the *Tabernanthe iboga* plant of Central West Africa which interacts with multiple receptor systems (Mash, 2023).

Despite their distinct pharmacological mechanisms, research suggests that these substances—particularly classic psychedelics and dissociatives—may exhibit convergent effects on brain functional connectivity (Gattuso et al., 2023).

#### Advances in psychedelic therapy research

During the mid-20th century, the clinical use of psychedelics gained momentum, particularly through two distinct therapeutic models: the psycholytic and psychedelic approaches. The psycholytic model involved repeated administration of low to moderate doses of psychedelics (e.g., LSD 25–200 μg) alongside psychodynamic psychotherapy to access unconscious material, enhance relational dynamics, and foster sustained psychological integration (Passie et al., 2022; Kishon et al., 2024). In contrast, the psychedelic approach utilized higher doses (e.g., LSD >200 μg) to induce transformative experiences, such as ego dissolution and mystical-type states, which were seen as catalysts for rapid psychological shifts. These peak experiences, often accompanied by emotional breakthroughs, were thought to directly elicit therapeutic change by disrupting entrenched patterns of thinking and behavior (Smith, 1958).

The psycholytic model’s iterative approach anticipated many principles now central to contemporary psychedelic therapy, particularly the importance of set and setting—concepts describing the psychological and physical context in which the drug is used (Hartogsohn, 2016). “Set” refers to the individual’s mindset, including mood, expectations, personality traits, and mental health, while “setting” encompasses the physical and social environment, such as location, social context, and cultural background (Hartogsohn, 2016). By meticulously managing the psychological and physical environment, psycholytic therapy fostered conditions that facilitated sustained personality changes through repeated, relationally focused sessions. The emphasis on facilitating psychological integration and relational dynamics would seem to resonate with this paper’s exploration of dissociative processes and their modulation through psychedelic use. Despite these promising developments, growing recreational use, safety concerns, and sociopolitical pressures led to the prohibition of psychedelics by the late 1960s and early 1970s (Kishon et al., 2024).

The contemporary landscape of psychedelic research is experiencing a resurgence often termed the “Psychedelic Renaissance” (Sessa, 2018). This revival reflects growing interest in the therapeutic potential of psychedelics, partly in response to the limitations of conventional pharmacological treatments for mental health conditions (Moncrieff et al., 2023). Recent clinical trials have demonstrated the potential efficacy of psychedelic-assisted therapies in treating various mental health disorders, including anxiety associated with life-threatening illnesses, major depressive disorder (MDD), and substance use disorders such as tobacco and alcohol dependence (Johnson et al., 2014; Morgan et al., 2017; Galvão-Coelho et al., 2021; Bogenschutz et al., 2022; Raison et al., 2023; Sloshower et al., 2023; von Rotz et al., 2023; Erritzoe et al., 2024). These trials typically administer psychedelics in controlled clinical settings, accompanied by supportive psychotherapy.

Ketamine, in particular, has gained attention for its rapid antidepressant effects at subanesthetic doses in patients with treatment-resistant depression (TRD) and bipolar depression, often surpassing conventional antidepressants in remission rates (Alnefeesi et al., 2022; Smith-Apeldoorn et al., 2022). In 2013, intravenous ketamine received Breakthrough Therapy Designation (BTD) for use in TRD from the US Food and Drug Administration (FDA), Later, in 2019, esketamine (“Spravato”), the (S)-enantiomer of ketamine, received FDA approval for adults with TRD, and in 2020, it was approved for adults with MDD and acute suicidal ideation or behavior (Thomas et al., 2016; Singh et al., 2020). Similarly, the FDA granted BTD to psilocybin in 2018 for TRD and in 2019 for MDD, based on clinical trials demonstrating a rapid and sustained antidepressant response (Carhart-Harris et al., 2016; Carhart-Harris et al., 2021; Davis et al., 2021; Goodwin et al., 2022; Metaxa and Clarke, 2024). More recently, in 2024, the FDA granted BTD to CYB003, a deuterated psilocybin analogue being investigated as an adjunctive treatment for MDD (Pharmacy Times, 2024).

Of particular relevance to this paper, the FDA granted BTD to MDMA-assisted therapy (MDMA-AT) in 2017 for PTSD treatment after Phase 2 trials demonstrated an acceptable risk–benefit profile (Mithoefer et al., 2019). In the landmark phase 3 MAPP1 trial, MDMA-AT was generally well-tolerated and significantly reduced PTSD symptoms, with a recent confirmatory study showing that 71.2% of participants receiving MDMA-AT no longer met DSM-5 criteria for PTSD, compared to 47.6% in the placebo-with-therapy group (Mitchell et al., 2023a; Mitchell et al., 2023b). Notably, however, the FDA has requested an additional trial to further evaluate MDMA-AT’s safety and efficacy for PTSD before granting full approval. MDMA-AT’s therapeutic effects are thought to arise through mechanisms that include promoting empathy, openness, and prosocial behavior; modulating fear memory reconsolidation; enhancing fear extinction; and reopening the critical period for social reward learning (Hysek et al., 2014; Wagner et al., 2017; Feduccia and Mithoefer, 2018; Nardou et al., 2019). These processes facilitate emotional engagement with therapy, enabling patients to reprocess traumatic memories and integrate their emotional impact in a more adaptive manner.

### Psychedelic iatrogenic structural dissociation: an exploratory hypothesis

In recent years, personal use of psychedelics has markedly increased, driven by growing research into their therapeutic potential and shifting perceptions of their risks, such as the decreasing perceived risk of LSD (Ko et al., 2023; Barnett et al., 2024b; Yockey et al., 2020). The progression of psychedelic research, along with shifts in legal classifications, indicates a continued upward trend in accessibility and use. As previously noted, research suggests numerous potential benefits associated with psychedelics, both in controlled clinical settings and beyond (Ko et al., 2023). However, alongside these promising developments, it is essential to explore potential risks, including the possibility of iatrogenic effects.

#### Evaluating psychedelic safety: contemporary findings and considerations

This section provides an overview of current evidence on the safety and potential risks associated with psychedelic use, setting the stage for later discussions on trauma-related dimensions of adverse effects. A recent review highlights that many risk perceptions associated with psychedelics originate from the first wave of psychedelic repression in the mid-20th century, largely fueled by sensationalized media reports that have perpetuated stigma (Schlag et al., 2022). However, decades of research and clinical experience support the safe administration of psychedelics under medical supervision, with increasingly refined guidelines for responsible use (Johnson et al., 2008; Gorman et al., 2021). Contemporary research largely refutes perceived harms such as addictive potential, neurotoxicity, and cardiotoxicity (Nichols, 2016; Nichols and Grob, 2018; Schlag et al., 2022). It is important to acknowledge, however, that ibogaine use has been associated with significant cardiac risks such as QT prolongation and Torsades de Pointes (TdP), particularly when used in unsafe settings without emergency medical care and advanced cardiac monitoring (Luz and Mash, 2021).

A 2022 study using data from the 2017 Global Drug Survey provides further support for the relative safety of psychedelics outside clinical settings (Kopra et al., 2022). Among 9,233 psilocybin mushroom users, only 19 (0.2%) sought emergency medical treatment within a 12-month period, primarily for psychological symptoms such as anxiety or paranoia (Kopra et al., 2022). The study highlights the importance of set and setting in shaping outcomes noting that adverse incidents were most often linked to unfavorable conditions, such as a negative mental state or a stressful environment (Kopra et al., 2022). A 2023 study by the same authors, using data from the 2020 Global Drug Survey, found similarly favorable safety outcomes, with only 0.9% of participants (*N* = 3,364) seeking emergency medical treatment after self-treatment with LSD or psilocybin mushrooms (Kopra et al., 2023).

However, it is noteworthy that 22.5% of the 3,136 respondents reported experiencing at least one acute adverse effect from self-treatment with psychedelics (Kopra et al., 2023). Research on these acute distressing experiences—often referred to as “bad trips”—has gained increasing attention, leading to the development of scales to assess their intensity. Such experiences are typically characterized by intense fear, confusion, and a perceived loss of control (Barrett et al., 2016; Johnstad, 2021; de Laportalière et al., 2023). Qualitative studies, however, indicate that many individuals ultimately view these challenging experiences as beneficial, often attributing them with existential insights (Gashi et al., 2021).

One study found that narratives of bad trips frequently involve “dramatic or even traumatic experiences” that remain partially unprocessed, with researchers suggesting that reframing these stories with humor or meaning can help individuals make sense of the experience (Sandberg et al., 2015; Gashi et al., 2021). This aligns with broader evidence on the role of narrative in trauma recovery, where the reconstruction or retelling of traumatic events is considered integral to the healing process (Kaminer, 2006; Herman, 2015). By narrating their experiences, psychedelic users may engage in a coping mechanism that allows them to process and integrate challenging experiences into their autobiographical memory. This sense-making through narrative may also explain why individuals continue to use psychedelics despite previous distressing episodes (Gashi et al., 2021).

The potential for psychedelics to induce psychosis, particularly in individuals with a personal or family history of psychiatric disorders, has long been a topic of speculation, though evidence remains limited (Strassman, 1984; Honk et al., 2024). A recent longitudinal study using representative samples from U.S. and U.K. adult populations found no significant increase in psychotic symptoms associated with psychedelic use, except in individuals with a personal or family history of bipolar disorder, where symptoms did increase. Interestingly, among individuals with a personal (but not familial) history of psychotic disorders, the study observed a decrease in the number of reported psychotic symptoms (Honk et al., 2024).

#### Enduring adverse effects following psychedelic drug use

Research on enduring adverse effects—those persisting long after the pharmacological effects of psychedelics have subsided—remains limited, and little is understood about the persistent psychological challenges that some users face post-experience.

A large internet survey by Carbonaro et al. (2016) aimed to characterize challenging experiences associated with psilocybin, focusing on participants’ most distressing “bad trip.” While many reported beneficial effects, 24% experienced adverse psychological symptoms lasting a week or longer post-use, and 10% reported symptoms extending beyond a year, with 7.6% seeking professional treatment for these effects (Carbonaro et al., 2016). Other large-scale studies have reported similar long-term effects, particularly following ayahuasca use. For instance, the Global Ayahuasca Project found that 55.9% of 10,836 participants reported adverse mental health effects in the weeks or months after use (Bouso et al., 2022). Despite this high incidence, approximately 88% of participants perceived these challenges as part of a positive therapeutic process, and the persistence of adverse effects did not deter most from continued participation in ayahuasca ceremonies. Factors associated with these effects included the intensity of the experience, pre-existing anxiety, and physical health conditions, while religious contexts of use were linked to fewer adverse outcomes (Bouso et al., 2022). In a sample of U.S. adults, Simonsson et al. (2023) found that 6.7% of classic psychedelic users reported thoughts or attempts at self-harm or harm toward others post-use, with 2.6% seeking medical or psychological assistance following their most distressing experiences.

Historically, the prevalence of persistent psychiatric conditions following psychedelic use has been considered lower than in the general population, and enduring mental health issues induced by these substances are rarely reported compared to other psychoactive drugs (Vis et al., 2021). Nonetheless, enduring adverse effects do occur, and concerns about their potential impact are increasingly relevant (Gable, 2007; Bouso et al., 2022). The risk of adverse effects is notably higher in sub-optimal settings, particularly in the absence of adequate guidance or psychological and spiritual support and may be especially pertinent for individuals with a history of trauma, with some evidence suggesting a link between childhood trauma and the onset of dissociative symptoms following classic psychedelic use (Thal et al., 2021; Perkins and Sarris, 2021). Furthermore, personality disorders, which are frequently associated with trauma, constitute another significant risk factor for adverse psychological events following psychedelic use (Yuan et al., 2023; Marrocu et al., 2024).

Hallucinogen Persisting Perception Disorder (HPPD) represents a debated long-term risk of psychedelic use (Grinspoon and Bakalar, 1979). While the precise mechanisms remain poorly understood, emerging evidence suggests that trauma and dissociation may play a significant role in its manifestation. Halpern et al. (2018) conceptualize Type 2 HPPD as a trauma-related disorder akin to PTSD, where persistent visual phenomena mirror intrusive recollections or dissociative flashbacks. Risk factors implicated in HPPD include co-occurring dissociative conditions and predispositions to depersonalization and derealization, suggesting that unresolved trauma may contribute to the development of perceptual disturbances (Holland and Passie, 2011; Halpern et al., 2018). Dissociation, strongly correlated with HPPD, may offer a partial explanatory model for its mechanisms, though further research is needed to clarify the mechanisms underlying HPPD and their implications for clinical care (Halpern et al., 2018).

#### A mixed methods study of extended difficulties following the use of psychedelic drugs

Recent research by Evans et al. (2023) employed a convergent mixed-methods approach to investigate “extended difficulties” following psychedelic experiences. The study included 608 participants who reported on the nature and duration of their challenges. The substances most commonly involved were psilocybin (27%) and LSD (25%), used either in social settings with friends or partners or in solitary contexts. Findings indicate that many participants experienced significant difficulties lasting from one to three years, with some challenges persisting beyond three years. Notably, a curvilinear trend emerged in the prevalence of extended difficulties, peaking in the first week post-use and again after one year.

Emotional challenges were the most frequently reported difficulty type, affecting 76% of participants, followed by challenges related to self-perception (58%), cognitive issues (52%), ontological concerns (50%), and spiritual struggles (34%). One noteworthy finding was that 40% of participants acknowledged a traumatic experience in childhood or adolescence, which they believed contributed to their post-experience challenges, suggesting a potential link between early trauma and the likelihood of acute adverse effects and prolonged difficulties with psychedelics.

To further explore the nature of these difficulties, the study analyzed participants’ written narratives, identifying several key themes that provide deeper insight into these experiences.

##### Emotional difficulties

Emotional difficulties were the most commonly reported challenge, cited by 67% of respondents. Resurfaced trauma was a significant aspect for 6%, with participants describing flashbacks and feeling overwhelmed by repressed traumatic memories, which they reported having significant difficulty processing and integrating. Additional emotional difficulties included declines in self-esteem, persistent negative intrusive thoughts, deepened self-shame, and feelings of powerlessness—all commonly associated with unresolved early childhood trauma.

##### Existential and ontological difficulties

Existential and ontological difficulties were reported by 42% of participants. *Derealization* emerged as a prominent sub-theme within this group, affecting 15% of those who experienced existential challenges. Participants expressed confusion and uncertainty about the nature of reality, often perceiving the world as surreal or “wrong” in the weeks and months following their experience. These issues frequently co-occurred with *depersonalization*, where individuals felt disconnected from their sense of self and surroundings, complicating efforts to integrate the psychedelic experience into daily life.

##### Self-perception difficulties

Approximately 23% of participants reported difficulties related to changes in self-perception and identity. *Depersonalization* was particularly common, affecting 16% of respondents. Participants described feeling disconnected from their bodies, experiencing a sense of self-disintegration, and struggling to relate to the person they were prior to the psychedelic experience. Additional sub-themes included a *diminished or disempowered sense of self*, marked by reduced self-esteem, and *excessive self-consciousness or self awareness* that participants found challenging to manage.

##### Perceptual difficulties

Perceptual difficulties were reported by 21% of participants, with the most prevalent sub-theme being *Visual Distortions / Hallucinations*, reported by 12% of respondents. These visual disturbances often resembled symptoms of HPPD, including visual anomalies such as visual snow, tracers, and heightened sensitivity to light. Additionally, five respondents reported experiencing prosopagnosia, a condition that impairs facial recognition and can significantly impact social interactions, often deepening feelings of disorientation and alienation.

Another commonly reported sub-theme was *Flashbacks* characterized by participants’ unsettling re-experiencing of elements of their psychedelic experiences. Flashbacks were occasionally triggered upon waking from sleep, leaving participants feeling disoriented and struggling to distinguish between the residual effects of their experience and the present moment. The study authors suggest that such flashbacks may represent a traumatic response, particularly when the original experience was perceived as distressing or overwhelming (Evans et al., 2023). One participant explicitly linked these flashbacks to a PTSD diagnosis.

Participants also described *Auditory Distortions/Hallucinations* as part of their perceptual difficulties. These auditory anomalies ranged from increased sensitivity to noise and distorted sounds, such as buzzing, to more severe cases of auditory hallucinations, including hearing voices.

The sub-theme of *Non-specific Sensory Distortions/Hallucinations* encompassed a broader array of sensory disruptions, including tactile, taste, and olfactory anomalies that persisted beyond the psychedelic experience.

Lastly, some participants reported an enduring distortion in their *Sense of Time*, with some noting that time seemed to slow down significantly, distorting their perception of events. This altered sense of time caused considerable stress and, in some cases, led participants to question their sanity, contributing to ongoing stress and low mood.

#### Case analysis of long-term negative psychological responses to psychedelics

In addition to Evans et al. (2023) and Bremler et al. (2023) conducted a study focusing on long-term negative psychological responses to psychedelics. This study, which employed a two-phase design, defined negative outcomes as self-perceived adverse psychological effects lasting at least 72 h post-psychedelic use. Participants were required to attribute these effects either entirely, mostly, or partially to the psychedelic substance they consumed.

In the first phase, researchers administered an online questionnaire to gather quantitative data from a broad sample, while the second phase involved semi-structured interviews with 15 participants from phase one. From the 32 survey completers, the researchers identified 18 recurring symptoms. Additionally, through thematic analysis of the interviews, three further symptoms—*derealization*, *disconnection (including perceived stigmatization)*, and *flashbacks of acute psychedelic experiences*—emerged, each reported by seven or more of the 15 interviewees (Bremler et al., 2023) (see Table 1).

**Table 1:** Summary of symptoms identified in Bremler et al. (2023) study on long-term negative psychological responses to psychedelics.

Symptom	Interview participants (*N* = 15)	Survey completers (*N* = 32)
Flashbacks	10 (66.7%)	N/A
Feelings of isolation/stigmatization	9 (60%)	N/A
Derealization	7 (46.7%)	N/A
Anxiety	14 (93%)	26 (81.3%)
Panic	13 (87%)	20 (62.5%)
Intrusive thoughts	12 (80%)	17 (53.1%)
Obsessive thoughts/behavior	11 (73.3%)	17 (53.1%)
Psychological distress	11 (73.3%)	17 (53.1%)
Sleep disturbances	8 (53.3%)	16 (50.0%)
Symptoms of depression	9 (60.0%)	15 (46.9%)
Complete loss of pleasure	9 (60.0%)	13 (40.6%)
Extreme/problematic distractibility	6 (40.0%)	11 (34.4%)
Abnormally high mood	4 (26.7%)	10 (31.3%)
Suicidal thinking/planning behavior	6 (40.0%)	10 (31.3%)
Visual disturbances	6 (40.0%)	8 (25.0%)
Paranoia	4 (26.7%)	5 (15.6%)
Delusional thinking	3 (20.0%)	5 (15.6%)
Addictive thoughts and behaviors	3 (20.0%)	5 (15.6%)
Auditory Hallucinations	2 (13.3%)	4 (12.5%)
Impulsive behavior	1 (6.67%)	3 (9.4%)
Self-Harm	1 (6.67%)	1 (3.1%)

Among the 15 interviewees, LSD was the most commonly used substance (7 participants), followed by MDMA (6 participants) and psilocybin (4 participants). Four participants reported simultaneous polydrug use, involving the consumption of multiple substances at once. A majority of interviewees (11 out of 15) described their acute psychedelic experiences as negative or frightening, all of which had used either LSD, psilocybin, or MDMA. In contrast, the remaining four participants, three of whom used MDMA (potentially with other stimulants) and one who used LSD, reported generally pleasant and positive experiences.

##### Flashbacks of acute psychedelic experience

Flashbacks were described by 12 of 15 interviewed participants (80%), with participants frequently using the terms “flashbacks” and “emotional flashbacks” to characterize their connection to a challenging experience. Eleven of these twelve participants had used a classic psychedelic and described the experience as overtly negative and/or frightening. Flashbacks were often triggered by environmental cues or reminders, though some participants experienced flashbacks without any discernible trigger. The onset of these symptoms varied widely: some participants reported flashbacks immediately after the experience, while others noted a delayed onset of up to two months later.

##### Disconnection (including sense of isolation/stigmatization)

Feelings of disconnection were reported by 7 interviewed participants (47%), encompassing experiences of isolation and a sense of separation from others. This theme included a profound sense of aloneness and social stigma, as illustrated by the following participant statements:

- Participant 10 (P10) described a “fundamental sense of aloneness,” expressing feelings of being beyond help: “No one can help me. I’m slipping into hell… my soul is alone; I’m completely disconnected from the universe.”
- Participant 4 (P4) highlighted social stigma, stating, “I could not talk to people about it because then you are just crazy.”
- Participant 26 (P26) emphasized the isolating nature of these experiences, remarking that “it’s not so much about having the symptoms; it’s the isolation.”

##### Derealization

Derealization was also reported by 7 interviewed participants (47%), who described experiences of detachment from their surroundings or a sense of unreality:

- Participant 20 (P20) reported “having daily out-of-body experiences.”
- Participant 3 (P3) expressed that “reality felt thin or unstable.”
- Participant 10 (P10) described feeling “completely disconnected from the universe.”
- Participant 23 (P23) specifically referred to their experience as “derealization,” explaining, “It was a… derealization… like there was something wrong with my normal world.”

For some participants, seeking professional support was hindered by the stigma associated with psychedelics. Three participants (P25, P3, P15) reported difficulty accessing mental health services, which compounded their feelings of isolation and disconnection.

An illustrative case is that of Participant 25 (P25), who described their LSD experience as positive overall but reported symptoms of HPPD as a lasting adverse response, primarily characterized by visual disturbances. Anxiety subsequently developed as a secondary symptom, driven by these visual disturbances.

Participant 22 (P22) recounted a particularly distressing experience in which they saw personally traumatic events from an out-of-body perspective during their experience. Although aware that these events had already occurred in their life, the external perspective intensified their fear, resulting in a prolonged period of anxiety, flashbacks, nightmares, and agoraphobia. In the interview conducted approximately twelve years after the event, P22 reported that most symptoms had subsided but noted significant, lasting changes in their sense of self and life perspective.

## Discussion

Recent research highlights that some individuals experience enduring psychological difficulties following psychedelic use, often associated with unresolved trauma (Evans et al., 2023; Bremler et al., 2023). This paper introduces the Psychedelic Iatrogenic Structural Dissociation (PISD) hypothesis, proposing that psychedelics may amplify access to dissociated traumatic material, increasing the risk of psychological destabilization in vulnerable individuals. Psychedelics, as “unspecific amplifiers” of mental processes, have the unique ability to bring unresolved trauma into conscious awareness in ways that can be profoundly disruptive (Bisbee et al., 2018; Grof, as cited in Stolaroff, 2022). For vulnerable individuals, this amplification may overwhelm coping systems, leading to destabilization or retraumatization. Such experiences resonate with Ivor Browne’s concept of “unexperienced experience,” in which trauma remains trapped in a “frozen present,” inaccessible to conscious processing until catalyzed by significant events like psychedelic experiences (Browne, 1990). However, when guided by trauma-informed support, these same processes present an opportunity for integration instead of destabilization, allowing individuals to engage deeply with dissociated material and move toward psychological healing. The discussion integrates the PISD hypothesis with existing evidence, identifying key risk factors and proposing strategies to enhance the safety and efficacy of psychedelic therapies.

### Applying structural dissociation to psychedelic use

The PISD hypothesis, grounded in trauma theory and applying structural dissociation theory, proposes that psychedelics may amplify access to unintegrated psychological material, exposing individuals to latent traumatic memories embedded within distinct personality parts. Structural dissociation theory posits that trauma disrupts the cohesion of personality, dividing it into an “Apparently Normal Personality” (ANP), responsible for daily functioning, and an “Emotional Personality” (EP), which governs survival responses and stores traumatic memories (Nijenhuis et al., 2010). When trauma-induced disruptions compromise integration between the ANP and EP, individuals may become vulnerable to psychological destabilization during and after psychedelic experiences (Van der Hart et al., 2004; Nijenhuis et al., 2010).

This framework aligns with evidence suggesting that individuals with early trauma histories are particularly susceptible to enduring adverse effects following psychedelic use. These difficulties often manifest as emotional dysregulation, identity fragmentation, existential distress, and unresolved feelings of guilt and shame. For example, Evans et al. (2023) found that 40% of participants reporting extended difficulties post-psychedelic use had childhood trauma histories. Similarly, Bremler et al. identified an association between trauma histories and persistent psychological symptoms, including anxiety, dissociation, and depression. Additional evidence from Thal et al. (2021) and Simonsson et al. (2023) further underscores the heightened vulnerability of individuals with childhood trauma to psychiatric symptoms and dissociative responses following classic psychedelic use. These findings align with previous research indicating that significant life events prior to psychedelic use may predispose individuals to enduring psychological difficulties (Kočárová et al., 2021).

For those with unresolved trauma, psychedelics may destabilize the delicate balance between trauma re-experiencing and daily functioning, increasing the risk of enduring psychological difficulties if the trauma remains unprocessed (Van der Hart and Rydberg, 2019). However, with trauma-informed support, amplified access to EP content during psychedelic experiences may facilitate trauma processing and integration, thereby offering a pathway toward psychological healing.

#### Emotional dysregulation and intrusive symptoms

Prolonged emotional dysregulation is among the most frequently reported adverse outcomes following psychedelic experiences, as noted in earlier studies (Evans et al., 2023; Bremler et al., 2023). Common difficulties include anxiety, panic, and distress, often linked to the resurfacing of unprocessed traumatic material. According to structural dissociation theory, unprocessed memories stored within the EP can resurface as sensorimotor and affect-laden experiences when triggered (Nijenhuis and den Boer, 2009). As the ANP becomes increasingly burdened, dissociation may intensify, perpetuating psychological instability and emotional distress.

Flashbacks emerged as a particularly distressing long-term symptom, with some participants describing spontaneous or triggered episodes resembling PTSD flashbacks. From the perspective of structural dissociation theory, flashbacks represent involuntary intrusions of unprocessed traumatic material stored in the EP into the ANP’s domain. This fixation on unresolved traumatic memories perpetuates re-experiencing, as the material remains unintegrated. Psychedelics may amplify conscious access to unprocessed EP material, leading to intrusive symptoms and prolonged distress in the absence of adequate therapeutic support. However, this process may also present opportunities for trauma integration. Notably, flashback phenomena have also been reported in recent clinical trials involving healthy participants (Müller et al., 2022).

#### Identity fragmentation and existential distress

Many individuals grapple with identity fragmentation and existential distress following psychedelic experiences. Participants in prior studies reported derealization, depersonalization, ontological confusion, and disruptions in self-continuity, often accompanied by a sense of alienation and instability (Evans et al., 2023; Bremler et al., 2023).

Recently, Argyri et al. (2024) further explored these phenomena in a study of 26 participants from the Evans et al. (2023) study, selected for their experiences of “Existential Struggle.” Participants reported pervasive fear, confusion, and encounters with ontologically challenging phenomena, such as perceived entities, extrasensory awareness, and out-of-body experiences, often leading to identity questioning, chronic derealization, and paranoia. These phenomena may reflect the EP’s fragmented representation of reality, which, when amplified, overwhelms the ANP’s capacity to maintain self-coherence. Notably, many participants in this study identified their experiences as traumatic, exhibiting PTSD-like symptoms such as re-experiencing, avoidance, mood disturbances, and functional impairment (Argyri et al., 2024). These disruptions may reflect the ANP’s difficulty integrating EP material, resulting in chronic dissociation and ontological uncertainty.

#### Guilt, shame, and evaluative conditioning

The pervasive feelings of guilt and shame reported by participants may be understood through evaluative conditioning—a process involving associative learning wherein neutral stimuli acquire negative emotional associations when paired with traumatic experiences (Nijenhuis and den Boer, 2009; Evans et al., 2023). For instance, in a shame-inducing event, the ANP may become conditioned to feel shame toward the EP, while the EP internalizes self-rejection and self-contempt. Evaluative conditioning may, therefore, exacerbate shame and guilt by deepening the ANP’s rejection of the EP, thereby reinforcing dissociative divides and hindering the integration of traumatic memories into a cohesive self-concept (Nijenhuis and den Boer, 2009).

### Implications for psychedelic therapy and research

Effective screening and preparatory protocols are crucial for identifying therapeutic interventions tailored to individuals with heightened vulnerability to adverse effects during and after psychedelic sessions. Incorporating validated dissociation assessment tools, such as the Dissociative Symptoms Scale (DSS), its shorter version, the Brief Dissociative Symptoms Scale (DSS-B), and the Dissociative Experiences Scale-II (DES-II), offers a robust framework for patient screening and monitoring in psychedelic therapy (Carlson et al., 2018; Saggino et al., 2020; Macia et al., 2023). These tools assess clinically significant dissociative symptoms—including amnesia, depersonalization, and absorption—across diverse domains. Integrating such measures into therapeutic protocols could enhance clinicians’ ability to identify individuals at risk of dissociative reactions and inform strategies for intervention both pre- and post-treatment.

Building upon this foundational screening, trauma-focused therapeutic approaches play a pivotal role in addressing dissociative vulnerabilities. Psychedelic Somatic Interactional Psychotherapy (PSIP), which engages both the autonomic nervous system and relational processing, is one such approach that has shown promise for individuals with complex trauma histories (Razvi and Elfrink, 2020). By emphasizing the therapeutic role of bodily sensations and interaction with the therapist, PSIP directly targets trauma that may be somatically stored. Its flexible use of directive and non-directive techniques allows therapists to adapt to patients’ evolving needs during psychedelic experiences, facilitating autonomic regulation and somatic processing. Preliminary studies are indicative of PSIP’s potential efficacy, with cannabis-assisted PSIP yielding positive outcomes in addressing relational trauma (Ragnhildstveit et al., 2023). Nearly all participants (22 out of 26) in a recent study by Argyri et al. (2024) identified body-oriented practices, such as yoga, acupuncture, breathwork, and trauma-release exercises (e.g., intentional shaking and vocal release) as essential tools for alleviating cognitive preoccupations associated with existential distress following psychedelic experiences (Argyri et al., 2024). These practices, noted for restoring awareness of the body and promoting emotional processing, align with PSIP principles (Forner, 2019). The present authors are preparing a follow-up paper to explore PSIP’s potential applications in greater depth.

Beyond somatic grounding, frameworks like ACER Integration, IFS, and research on spiritual, existential, and theological components offer complementary support for individuals grappling with post-experience challenges (Watts and Luoma, 2020; Palitsky et al., 2023). IFS, for instance, addresses the interplay of distinct parts of the self, providing a framework for patients to contextualize and reconcile dissociative experiences. Similarly, incorporating an intelligible metaphysical framework, as Sjöstedt-Hughes (2023) suggests, may help patients contextualise and make sense of profound metaphysical experiences, potentially enhancing therapeutic outcomes by aligning therapeutic integration with meaning-making processes. These approaches, by addressing both psychological and existential dimensions, could facilitate more comprehensive trauma integration.

Social support is another critical component in mitigating trauma-related risks and fostering recovery. Research highlights its ability to create a sense of safety, promote emotional and physiological regulation, and facilitate integration between the ANP and EP (Elklit, 1997; Runtz and Schallow, 1997; Nijenhuis and den Boer, 2009). Conversely, the absence of such support may exacerbate dissociative symptoms, as evidenced by individuals who report pervasive feelings of isolation and derealization following psychedelic experiences (Bremler et al., 2023; Evans et al., 2023). When significant others or social networks fail to acknowledge and assist with traumatic experiences, dissociative symptoms are likely to intensify (Nijenhuis and den Boer, 2009). Structured social support—through therapeutic alliances, peer networks, and stigma-free environments—can safeguard against these adverse outcomes by providing necessary relational support and fostering integrative capacity—the ability to process and synthesize traumatic experiences into a cohesive self-narrative (Nijenhuis and den Boer, 2009; Van der Hart and Rydberg, 2019; Boehnke et al., 2023). Emotional support, such as affirming presence or gentle touch from a trusted individual, has also been shown to stabilize psychophysiological responses during exposure to threatening stimuli, further enhancing trauma recovery (Nijenhuis, 2004).

Trauma-informed strategies provide a comprehensive framework for refining clinical trial methodologies and therapeutic protocols. Adaptive trial designs tailored to participants with complex trauma histories could improve the reliability of findings, while integrating structural dissociation theory into clinical protocols may address participant vulnerabilities more effectively. Standardizing these practices across clinical and research contexts could advance the field while ensuring participant safety remains central. Beyond research contexts, these strategies may have implications for regulatory guidelines and therapist training programs.

Future research should investigate the longitudinal efficacy of trauma-informed psychedelic therapies, with particular attention to their effectiveness across diverse demographic and clinical populations. Studies could explore the mechanisms through which interventions such as PSIP, IFS, and body-oriented practices mitigate dissociative symptoms and promote trauma integration. Additionally, further examination of validated dissociation assessment tools, including their predictive utility and cross-cultural applicability, is warranted to enhance screening protocols. Empirical research into the role of structured social support networks and the integration of intelligible metaphysical frameworks in psychedelic therapy could also illuminate pathways to optimizing therapeutic outcomes and reducing dissociative risks.

#### Neurophysiological mechanisms underlying PISD: insights from canalization, TEMP, and REBUS

Recent research provides valuable insights into the neurophysiological mechanisms that may underlie the PISD hypothesis, particularly through the constructs of canalization, plasticity, and predictive processing (Carhart-Harris et al., 2019; Carhart-Harris et al., 2023). These frameworks offer a deeper understanding of how psychedelics might interact with entrenched dissociative patterns, either destabilizing or facilitating their integration.

The concept of canalization, as introduced by Waddington (1959) and developed in contemporary psychopathology models, describes how rigid cognitive, behavioral, and neurobiological patterns emerge and persist as adaptive responses to adversity or distress. Over time, these patterns become entrenched, constraining psychological state space and diminishing the brain’s capacity for plasticity—the ability to reorganize and adapt in response to new information or experiences. While such canalized responses may initially serve to minimize uncertainty in threatening environments, they can become maladaptive, resisting revision even when environmental conditions change (Carhart-Harris et al., 2023).

This dynamic may be particularly relevant to trauma-induced division of the personality into an ANP and EP. Entrenched dissociative patterns—such as those protecting against traumatic memories stored in the EP—can be viewed as canalized states, rigidly maintained to avoid overwhelming distress. These states align with the defensive responses observed in PTSD and DD, where over-precise beliefs and behavioral strategies dominate psychological functioning. The cand plasticity model posits that psychedelics may counteract such canalization by increasing Temperature of Entropy Mediated Plasticity (TEMP). TEMP involves a transient increase in brain entropy—a state characterized by greater neural complexity and dynamic variability—allowing the system to escape entrenched cognitive and behavioral ruts. This process, akin to simulated annealing, facilitates the exploration of alternative psychological states and recalibration of the free-energy landscape, potentially allowing for the integration of dissociated material (Kirkpatrick et al., 1983; Carhart-Harris et al., 2023).

Building on these principles, the Relaxed Beliefs Under Psychedelics (REBUS) model offers a mechanistic explanation for how psychedelics disrupt entrenched patterns (Carhart-Harris et al., 2019). REBUS posits that psychedelics reduce the precision-weighting of rigid top-down predictive models, or hyperpriors, which normally constrain perception and behavior. This relaxation of priors facilitates the emergence of bottom-up sensory information, enabling suppressed material—such as unresolved trauma stored in the EP—to surface into conscious awareness (Carhart-Harris et al., 2019). From the perspective of the PISD hypothesis, this mechanism highlights the dual-edged potential of psychedelics. While loosening entrenched priors can promote psychological flexibility and integration, it may also destabilize individuals with significant trauma histories by overwhelming their coping systems. The amplified access to unprocessed EP content can lead to re-experiencing, emotional dysregulation, and identity fragmentation if not supported by trauma-informed therapeutic interventions.

Neuroimaging studies provide empirical support for the relevance of the canalization and REBUS models to the PISD hypothesis by demonstrating how psychedelics modulate brain connectivity and activity patterns associated with trauma processing. For example, MDMA-assisted therapy has been shown to alter connectivity in regions implicated in trauma processing, such as the amygdala, hippocampus, and Default Mode Network (DMN) (Singleton et al., 2023). Changes in amygdala-hippocampal connectivity, alongside reduced activation in DMN regions, may reflect a neurophysiological basis for enhanced integration of EP content (Singleton et al., 2023). These findings seem consistent with structural dissociation theory’s assertion that EP-stored trauma-laden memories become hyperactivated when integrative capacity is compromised (Van der Hart and Rydberg, 2019). Additionally, evidence of synaptic replenishment—such as increased dendritic spinogenesis following psychedelic use—provides further support for how psychedelics may reverse canalization and enhance neural flexibility (Shao et al., 2021). Future research could investigate whether these neurophysiological changes can serve as biomarkers for dissociative vulnerability and therapeutic efficacy, particularly in populations with trauma histories. Such studies could guide the development of trauma-informed psychedelic protocols tailored to enhance integrative capacity and reduce the risk of adverse outcomes.

Heightened context sensitivity, a central tenet of the REBUS model, is reflected in the variability of reported outcomes following psychedelic use, particularly regarding extended difficulties. For example, Evans et al. (2023) observed that certain adverse effects common in other studies were less prevalent in their sample, suggesting that contextual factors may significantly influence outcomes. Specifically, 8% of survey respondents reported extended difficulties following participation in a clinical trial or therapeutic session, indicating that these challenges are not confined to unregulated or informal settings. These findings underscore the importance of understanding how specific contexts—such as ceremonial, recreational, or clinical use—contribute to distinct patterns of extended difficulties (Evans et al., 2023). Future investigations could explore how these contextual dynamics interact with the neurophysiological processes described by REBUS and TEMP to influence integration or destabilization (Carhart-Harris et al., 2019; Carhart-Harris et al., 2023).

### Limitations

This paper presents the exploratory PISD hypothesis, proposing a novel framework linking prolonged psychological difficulties following psychedelic use to structural dissociation. However, several limitations should be noted. First, the analysis relies on secondary data, which is subject to inherent constraints such as small sample sizes, potential recall bias in self-reported data, and a lack of longitudinal tracking of extended difficulties. Additionally, the framework remains speculative, as no direct empirical validation of the PISD hypothesis has yet been conducted. The correlational nature of the findings limits their generalizability and precludes causal conclusions.

Structural dissociation theory has not been widely applied in psychedelic research, warranting further empirical studies to assess its relevance and refine its integration into this context. Broader research using diverse samples across cultural and clinical settings is essential to evaluate the hypothesis and establish its applicability to psychedelic therapies.

## Conclusion

The hypothesis of Psychedelic Iatrogenic Structural Dissociation (PISD) offers a novel framework for understanding how psychedelics may amplify dissociative processes in trauma-exposed individuals. While psychedelics hold immense therapeutic promise, their effects are highly context-dependent, with unresolved trauma potentially increasing the risk of long-term psychological difficulties. By integrating structural dissociation theory with proposed neurophysiological models, this paper explores mechanisms by which psychedelics may recalibrate entrenched dissociative patterns, offering opportunities for trauma integration while posing risks of destabilization. The findings underscore the critical need for trauma-informed approaches, including rigorous screening tools, personalized preparation protocols, structured integration practices, and robust social support systems, to enhance safety and efficacy. Future research could empirically validate the PISD hypothesis, refine trauma-informed protocols, and explore how contextual factors influence therapeutic and adverse outcomes. By advancing our understanding of dissociation and trauma integration in the context of psychedelics, the PISD framework can serve as a foundation for safer and more effective applications, maximizing the therapeutic potential of these substances while protecting trauma-exposed populations from long-term harm.

## Data Availability

The original contributions presented in the study are included in the article/supplementary material, further inquiries can be directed to the corresponding authors.
